# Sequence- and Interactome-Based Prediction of Viral Protein Hotspots Targeting Host Proteins: A Case Study for HIV Nef

**DOI:** 10.1371/journal.pone.0020735

**Published:** 2011-06-28

**Authors:** Mahdi Sarmady, William Dampier, Aydin Tozeren

**Affiliations:** Center for Integrated Bioinformatics, School of Biomedical Engineering, Science, and Health Systems, Drexel University, Philadelphia, Pennsylvania, United States of America; University of Kansas Medical Center, United States of America

## Abstract

Virus proteins alter protein pathways of the host toward the synthesis of viral particles by breaking and making edges via binding to host proteins. In this study, we developed a computational approach to predict viral sequence hotspots for binding to host proteins based on sequences of viral and host proteins and literature-curated virus-host protein interactome data. We use a motif discovery algorithm repeatedly on collections of sequences of viral proteins and immediate binding partners of their host targets and choose only those motifs that are conserved on viral sequences and highly statistically enriched among binding partners of virus protein targeted host proteins. Our results match experimental data on binding sites of Nef to host proteins such as MAPK1, VAV1, LCK, HCK, HLA-A, CD4, FYN, and GNB2L1 with high statistical significance but is a poor predictor of Nef binding sites on highly flexible, hoop-like regions. Predicted hotspots recapture CD8 cell epitopes of HIV Nef highlighting their importance in modulating virus-host interactions. Host proteins potentially targeted or outcompeted by Nef appear crowding the T cell receptor, natural killer cell mediated cytotoxicity, and neurotrophin signaling pathways. Scanning of HIV Nef motifs on multiple alignments of hepatitis C protein NS5A produces results consistent with literature, indicating the potential value of the hotspot discovery in advancing our understanding of virus-host crosstalk.

## Introduction

This study presents a bioinformatics approach to predicting hotspots on viral proteins mediating transient binding interactions with host proteins. The data used for these predictions consists of large collections of viral and host protein sequences and drafts of protein interactome maps between the virus and the host. Predictions are compared for validation with experimental data on the binding site predictions of Human Immunodeficiency Virus (HIV) Nef protein to host proteins. A hotspot is defined as a continuous protein sequence segment, 5 to 15 amino acids long, containing multiple short linear motifs [1] used by the viral protein to bind to different host proteins. Molecular dynamics [2], [3] and binding assay studies [4] indicate the presence of binding interface incidences between a short linear sequence segment of a protein and a structural topology on the opposing protein in a pair of binding proteins. The sequences interacting with the same (or similar) topology within a set of conditions is expressed as a regular expression [5]. The sum of such sequences comprises a short linear motif [6]. Hundreds of short linear motifs have been established as instrumental in mediating transient binding between proteins [7], [8]. In our definition, a hotspot contains both the core motif and the flank elements providing context for the specificity of the binding [3], [9], [10] and is a concept useful in comparing binding sites across viruses.

Viral infections of the human constitute a global public health problem. Hundreds of millions of people worldwide are infected with the Hepatitis B (HBV) and/or C Virus (HCV), viral roots of a chronic infection that in some cases leads to cirrhosis and liver cancer. Similarly, HIV infection continues to be a worldwide epidemic. Combination antiretroviral therapies against HIV have been successful in retarding the progress of the infection, however, these treatments are expensive and still not available to vast majority of HIV Positive individuals [11]. The poor performance of these drugs in some individuals, possibly due to acquired resistance, is a reason for the ongoing research for discovery of new AIDS drugs and HIV vaccines. Influenza is yet another viral epidemic with heavy toll in the human population. Considering the fact that a number of cancer subtypes such as cervical [12] and liver cancers [13], [14] have viral roots, it is important to identify host proteins targeted by viral proteins in outlining the progression of the infection.

Protein binding interactions between viruses and host have been investigated in recent years both experimentally [15], [16], [17] and computationally [18], [19], [20], [21], [22], [23], [24], [25], [26]. The experimental studies involve a multitude of binding assays with noisy outcome and computational studies use such data along with other molecular databases, and utilize tools of system modeling, machine learning and network analysis to arrive at new predictions or better annotations of existing draft networks of virus and host proteins. It is also clear from the result of the afrementioned studies that accurate predictions of virus-host protein interactions would benefit greatly from better understanding of the types of interface viral proteins make with host proteins.

One mode of of transient interaction between a virus protein and a host protein involves coupling of linear motifs on viral protein with a binding topology on the host protein [8], possibly at mutiple sites [23]. Whereas the short linear motifs are continuous segments of the protein sequence, the topology on the opposing protein may be composed of amino acid residues not necessarily adjacent in sequence and could be parts of the closely packed regions of proteins called domains. Because viral proteins are relatively flexible and contain disordered segments, they may potentially express motifs targeting host proteins. However, previous studies point to the shortcomings of existing collection of linear motifs and motif-topology correspondence annotations in accurately representing known interactions between HIV proteins and the proteins of the host [23], [25]. Recently emerging algorithms in the discovery of short linear motifs [1], [3], [27], [28] and associated computational approaches for probing motif-topology pairings [2], [29], [30] provide a valuable opportunity to increase our knowledge on the grammar of virus-host crosstalk. Detailed knowledge of binding interface between viral and host proteins will likely impact the speed of discovery of new antiviral drugs targeting both viral and host targets [31], [32], [33], [34].

In this study, we present a bioinformatics approach to identify potential binding sites on viral proteins targeting specific host proteins. We use motif discovery tools developed by others [28] in a novel way on collections of sequences of both viral and host proteins. Specifically, we consider known host targets of HIV Nef and for each such target we identify its binding partners using an interactome database [35]. We run the motif discovery algorithm on set of sequences of binding partners as well as multiple sequences of the HIV Nef. We consider from the resulting motif sets only those that are highly statistically enriched among the neighbors of Nef targeted host proteins in comparison with host protein sets with known interactions. Results indicate the value of the approach in predicting viral sequence motifs on relatively structured segments of the protein sequence. The system approach to predicting binding site maps of viral proteins will potentially facilitate identification of binding partner topologies using new computational appraches [2] as potential targets for drug dicovery.

## Results

### Approach

**Figure 1:**
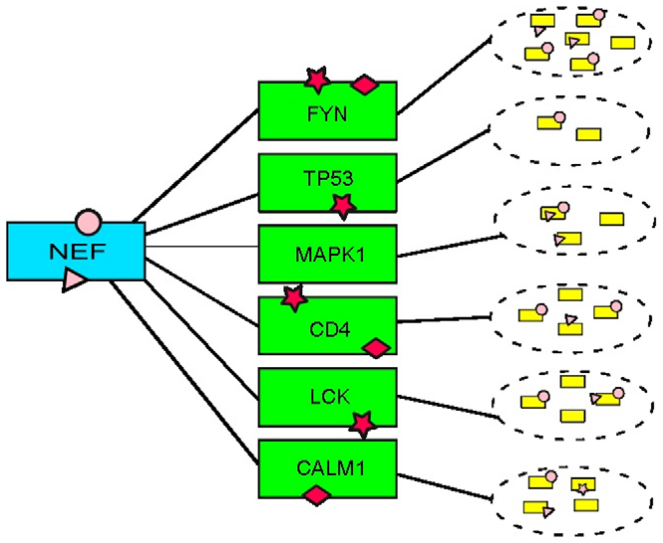
Schematic model of HIV Nef and its host targets. The figure shows a subset of host proteins known to be directly binding to Nef in green boxes (H1 proteins). The immediate binding partners of such host proteins (H2 proteins) are shown in smaller yellow boxes within ellipses drawn in dashed lines. Small pink geometrical shapes represent motifs commonly expressed by Nef and H12 and proteins. Red shapes are counter-motifs on H1 proteins.

Our approach is described in simple terms by using the following terminology. Let V represent a viral protein and H1 represent a host protein (or set of host proteins) targeted by V for transient binding interactions. The binding partners of H1 proteins in the host proteome are represented by the symbol H2. Within the H2 set, some proteins will be outcompeted by the viral protein V in their binding interactions with H1. We denote such proteins by the symbol H12. H12 proteins are assumed to share a set of linear motifs with V and that such motif combinations are highly enriched among H2 proteins in comparison to host protein sets with at least one known binding partner. A viral protein can potentially hijack function and outcompete H12 host proteins for binding to a targeted H1 host protein using motifs also expressed by H12 proteins [21]. In the presence of high viral loads within a host cell, there will be many more copies of viral proteins compared to number of copies of H12 proteins. Our computational recipe is as follows: given an H1 protein, we use the set of sequences of its H2 proteins plus multiple sequences of the viral protein V in executing the motif discovery operations (Figure 1). Preliminary experiments showed that addition to the set copies of viral protein sequences assured discovery of motifs conserved on viral sequences. Shown in Figure 1 is an illustration of this process where the viral protein under consideration is HIV Nef. H1 proteins are shown in green boxes identified by their gene symbol. The dashed lines represent binding partners (H2 proteins). The motifs shared by V and H12s are shown in pink symbols and their counter binding sites on H1s using red symbols. Our approach may be of questionable value in cases where a viral protein such as HIV Nef brings together two host proteins in an aggregate, effectively creating a new edge in the host protein network [36]. It is possible in such situations that viral proteins use motifs rarely used in the host proteome for binding interactions.

### Correspondence matrix for HIV Nef – host protein interactions

**Figure 2:**
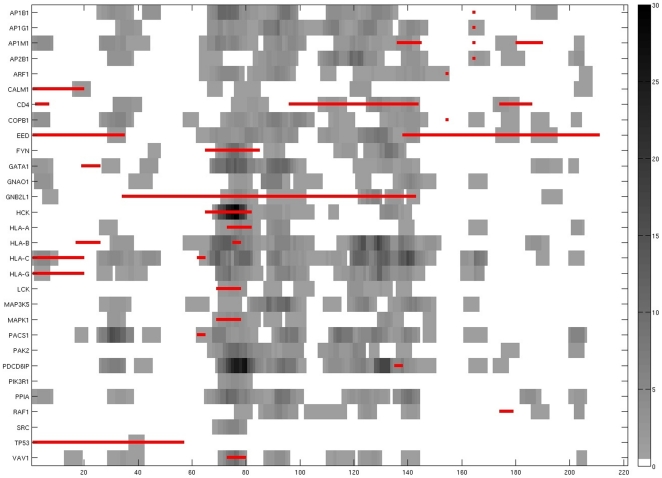
Correspondence matrix for HIV Nef – host protein interactions. The hotspots on the Nef sequence (horizontal axis) for binding are shown against the host targets (vertical axis). The gray scale intensity at an amino acid residue is proportional to number of motifs intersecting a residue. The white color indicates absence of hotspots. The horizontal red straight lines indicate experimentally identified Nef binding sites for each H1 protein.

The HIV-1, Human Protein Interaction Database (HHPID) identifies 42 host proteins undergoing direct binding interactions with HIV Nef including transient interactions that occur in phorphorylation and other modification events (Table 1) [17]. Shown in Table 1 are the numbers of binding partners of Nef targeted host proteins and the upper bound for the number of outcompeted H12 proteins for each H1, predicted by our analysis. We ran the motif discovery algorithm [28] only on those H1s with at least 10 binding partners. The output of the motif discovery step was a list of regular expressions of motifs aligned on viral protein sequence with high information content and significant p value (Table S1), indicating they are statistically enriched among H2 proteins. As shown in Figure 2, many such motifs clustered on a finite number of hotspots. The horizontal axis in Figure 2 indicates the distance along the Nef sequence in the units of number of residues, starting from the N terminal. The vertical axis lists the gene symbols of H1 proteins known to interact with Nef. The white area of the figure represents no motifs whereas the gray scale is proportional to the number of motifs containing the residue at a given position on the sequence. Also shown in the figure, in red horizontal lines, are the experimentally determined binding sites or binding regions on Nef for specific H1 proteins. As described in the methods, we gathered the experimental data from the summaries of HIV protein interactions given for each H1 protein at the NCBI gene web resource. The figure attests to the incompleteness of such data, as we found no binding sites for six of the thirty H1 proteins under consideration. In other cases, as in TP53, experimental studies provided a region of the sequence where potential binding sites existed. The figure indicates a distribution of predicted Nef binding motifs favoring the core region (Figure 2), possibly indicating the inefficiency of our prediction approach in detecting binding mechanisms at highly flexible loop regions of Nef.

**Table 1:** Human proteins targeted by HIV Nef in binding interactions tabulated in HHIPD database.

Symbol	Neighbors	Connectivity	Cancer SAM	Symbol	Neighbors	Connectivity	Cancer SAM
TP53*	266	68	Kidney, Prostate	COPB1	17	12	
SRC*	208	98		AP1B1	17	12	
MAPK1	160	65		AP1G1	17	13	
FYN	154	72		EED	17	11	
PIK3R1*	128	53	Breast, Skin	HLA-C	14	10	Liver
CALM1	114	49		HLA-B	14	13	Liver
LCK	105	42		PPIA	13	9	
RAF1	93	42		HLA-G	10	7	Liver
VAV1	62	31		PACS1	10	8	
GNB2L1	59	31		GATA3	8		Kidney, Skin
HCK	56	35	Adrenal	AP3D1	8		
PAK2	42	23		TNIP1	8		
ARF1	39	20		HLA-E	7		
MAP3K5	38	20	Adrenal	AP1G2	6		
CD4	33	16		CD86	5		
GNAO1	33	16	Brain, Liver	HLA-F	5		Liver
AP2B1	32	18		AP1S1	5		Adrenal
HLA-A	22	12	Liver	CD80	4		
AP1M1	21	12		AP1S2	3		
PDCD6IP	19	12		ATP6V1H	2		
GATA1	18	13		PSMB4	2		Cervix, Lung

List of host proteins binding to HIV Nef is presented along with (a) the number of binding partners in the host proteome as identified by the HPRD Database and (b) the number of H12 neighbors potentially outcompeted by HIV Nef. Host proteins targeted by both HIV Nef and HCV NS5A proteins are marked by the symbol *.

### System level comparison with experimental data

In order to assess validity of our predictions within the context of experimental binding site data presented in HHPID [17], we used two different statistical methods on the significance of the intersection of predicted hotspots with experimental data. First, we used hypergeometric test with Nef sequence length, and lengths of predicted sites, experimental sites and their intersection as inputs. Second, we used a much more complex statistical approach, namely the Hopkins-Skellam Index (HSI) [37], which takes into account of the spatial distribution of predicted and experimental binding sites on the Nef sequence. The p value computations using these two statistical approaches are presented in Table 2 for the predicted and experimental binding sites shown in Figure 2. The table shows hypergeometric test giving statistical significance to predicted binding sites for thirteen H1 proteins, eight of which also appear in the column for HSI predictions. These eight H1 proteins consist of CD4, a receptor for the HIV virus, membrane kinase FYN, G protein GNB2L1, Src family tyrosine kinases HCK and LCK, HLA-A, MAPK1, and GTP binding VAV1. The match between predicted and experiment is significant for vesicular trafficking associated ARF1, TP53, PDCD6IP, PACS1, EED, CALM1, AP1G1, and AP2B1 in the less stringent hypergeometric test. Hardly any experimental binding site data exists for H1 proteins PIK3R1, PPIA, RAF1, SRC, PAK2, and GNAO1. Mismatch between predicted and experiment is more frequent in cases where experimental binding sites fall onto flexible, disordered, loop-like regions of Nef.

**Table 2:** Statistical match between predicted and experimentally annotated HIV Nef binding sites to H1 proteins.

H1 proteins	p HPGT	p HSI
TP53*	0	
SRC*	X	X
MAPK1	0	0.0001
FYN	1.5 E - 6	0.0001
PIK3R1*	X	X
CALM1		
LCK	1.3 E - 7	0.0014
RAF1		
VAV1	0	0.0001
GNB2L1	4.6 E - 8	0.0001
HCK	2.6 E - 9	0.0001
PAK2	X	X
ARF1	0	
MAP3K5	X	X
CD4	9.8 E - 9	0.0008
GNAO1	X	X
AP2B1	0	
HLA-A	0	0.0001
AP1M1		
PDCD6IP	0	
GATA1		
COPB1		
AP1B!		
AP1G1	0	
EED		
HLA-C		
HLA-B		
PPIA	X	X
HLA-G		
PACS1	0	

The second and third columns list p values for hypergeometric test and Hopkins-Skellam index, respectively. P values >0.05 is not shown in the table. The symbol X refers to H1s with no experimental binding site data.

Our computations presented in Figure 2 suggest that HIV Nef interacts with an H1 protein typically in multiple hotspots rather than have a single point of contact. The employment of multiple sites for transient interaction of protein pairs is not unusual; it has been observed in a number of binding events such as phosphorylation [38] and in double-headed myosin-actin interactions during skeletal muscle contraction [39]. The Figure 2 also shows that Nef segments predicted to bind to different host proteins overlap with each other. Predicted binding sites form clusters along the core region of Nef (60–157) and less so in the N terminal region (0–59) and along the flexible loop (157–175) [40]. The hotspot at 71–77 is known to be part of the binding site to HCK SH3 domain to HIV Nef [41]. The Nef binding site to HCK interface is composed of a total buried surface of 1,264 Å2 formed by a proline-rich region (Nef residues 71–77) and the RT loop binding region involving hydrophobic key residues, Phe-90, Trp-113, and Ile-114, and a salt bridge involving Arg-77 [32]. Consistent with our Figure 2, a compound targeting this Nef site was shown to reduce binding to SH3 as well as MCH molecules but had no effect on CD4 binding [32].

### HIV Nef hotspots, their dependence on viral subtype and intersection with Nef epitopes

**Figure 3:**
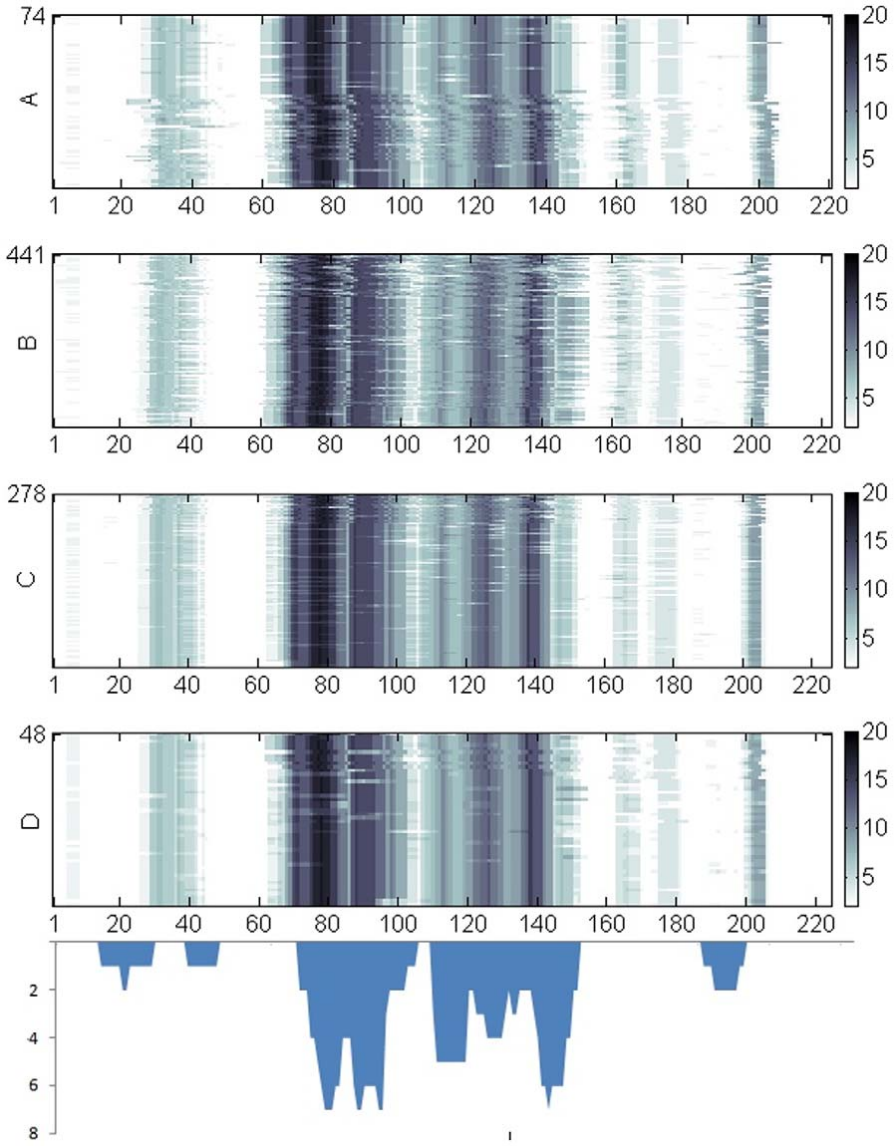
Amino acid sequence positions of motif hotspots on HIV Nef. The hotspots are shown on the horizontal axis (separated by subtype). Color intensity is proportional to the number of Nef targeted proteins with enriched hotspot motifs among their immediate neighbors. The histogram below shows the number density of well-defined CTL/CD8 epitopes present on the HIV Nef sequence.

HIV Nef hotspots appear independent of the virus subtype in our computations. This observation stems from our computation of hotspots for each of the four major subtypes of HIV-1 shown in Figure 3. The figure indicates hotspots along multiple sequence alignments of HIV Nef in four subtypes (A, B, C, and D). The gray scale intensity is proportional at any amino acid position to the number of H1's having a motif intersecting the amino acid residue. Because HIV Nef is polymorphic in length (200–215 amino acids) with the most common length being 206 [42], spatial positions of HIV Nef motifs vary on the thousands of sequences available in public domain. Shown in the figure are the alignments of approximately 50 percent of the sequences where motifs were conserved spatially without resorting to adding spaces between amino acids to achieve optimal spatial conservation. The observation that hotspots are largely conserved on four major subtypes of HIV (A, B, C, and D) may indicate their participation in the functional roles of Nef on HIV infectivity.

**Figure 4:**
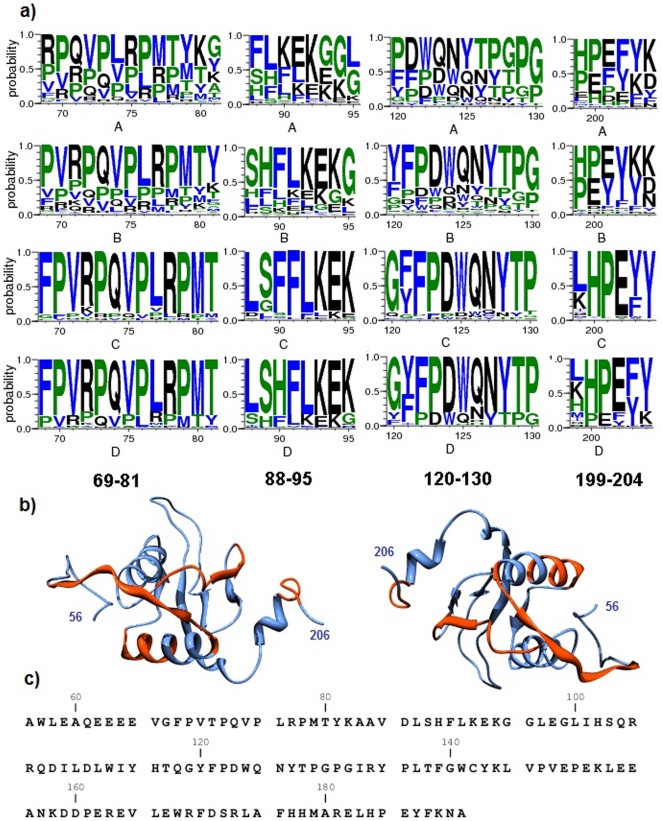
Sequence logos of HIV Nef hotspots containing the highest numbers of motifs. **a**) Sequence logos of four hotspots on Nef separated by subtypes; b) Regions covered by hotspots highlighted in red on 3D structure of Nef visualized from the PDB structure 2NEF [63]. Numbers on the Nef structures identify the start and end amino acid position of the 3D structure relative to the reference sequence of Nef. Molecular graphics images were produced using the UCSF Chimera public access software package [64]; c) Sequence of the Nef 3D structure shown in part b of the figure.

Also shown in Figure 3 is the spatial distribution of HIV Nef CTL/CD8 epitopes along the sequence of Nef. The epitopes and the hotspots closely match each other along the core region of Nef. This region contains HIV Nef binding sites for host proteins involved in immunity such as HLA-A and CD4 as well as binding sites for kinases and G proteins [17] (Table 2). HIV Nef epitopes and hotspots for CD4 and AP1M1 intersect at the N terminal but do not match with hotspots along the sequence segment (185–225). However, Nef peptides most frequently recognized by T cells of HIV-1-infected individuals were shown to be along 90–97, 135–143, 71–81, 77–85, 90–100, 73–82, and 128–137 [43], closely matching our predicted hotspots. In fact, as predicted in our computations, the HIV Nef 73–82 epitope was previously shown to bind strongly to HLA-A3 molecules [44]. The high immunogenetic property of the HIV Nef core region is reflected by the relative stability of the Nef sequence in this region [45]. This point is illustrated in Figure 4 in the form of sequence logos falling into the core region of Nef. There is little variation in sequence in these hotspots for HIV subtypes C and D. Note also that these hotspots partially intersect secondary structures (alpha helices and beta sheets) in the core region of Nef, indicating that motifs that make up these hotspots are not completely disordered, consistent with previous findings by Stein [3]. As shown in Table S1, hotspots in the core region include motifs for binding to a large number of host proteins and as such Nef sequence segment in this region maybe under environmental pressure for selected variation. The same table shows that many of the motifs that fall into hotspots in Figure 4 are either identical or highly similar to some of the motifs in the ELM database [46]. Similarity of regular expression of motifs discovered in this study to motifs in the literature facilitates functional annotation of the discovered motifs in terms of their function in events such as phosphorylation, sorting and internalization.

### Host proteins outcompeted by HIV Nef

We computed an upper bound for host proteins outcompeted by HIV Nef by selecting from H2 proteins those sharing the annotated Nef motifs. This set (H12 set) contained 572 human proteins among which 45 appeared in the HHPID as indirectly interacting with Nef. HHPID cites 300 host proteins as directly or indirectly interacting with Nef. The intersection of these two sets gives a p value equal to zero in hypergeometric test computed with respect to all Human Protein Reference Database (HPRD) proteins. Many of the host proteins indirectly interacting with HIV Nef may be further away from the Nef targeted H1 protein along a pathway, and therefore, one would not expect our predictions to contain all Nef interacting proteins. It is possible to further refine the estimate of outcompeted proteins by requiring such proteins contain at least two non-intersecting Nef motifs with 3D alignment procedures supporting the choices, and will be considered in a future study.

**Figure 5:**
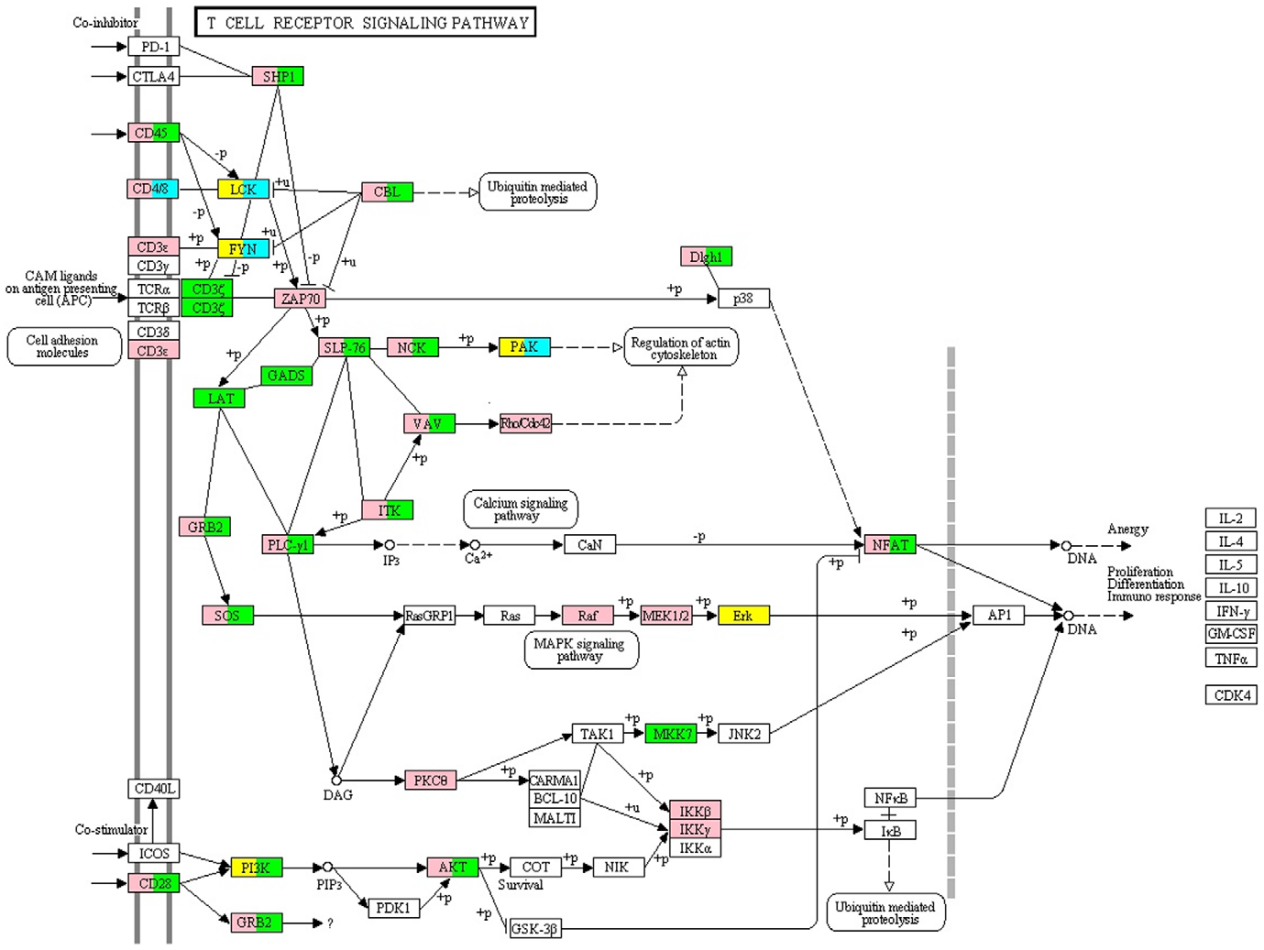
HIV Nef targets and potantially outcompeted proteins marked on KEGG T cell receptor pathway. The figure shows the host targets interacting with Nef hotspots spanning from position 69 to 81 and 120 to 130 of the Nef sequence. Yellow and cyan represent proteins targeted by hotpots 69–81 and 120–130, respectively. Pink and green represent proteins potentially outcompeted by Nef via its interaction through its 69–81 and 120–130 hotspots, respectively.

Next, we considered the statistical enrichment of KEGG [47] pathways with H1 and H12 proteins. Results shown in Table 3 indicate highly enriched pathways (p<10E-15) including the T cell receptor pathway, natural killer cell mediated cytotoxicity, ERB and insulin signaling pathways, and pathways in cancer. We have marked on T cell receptor pathway those H1 proteins that bind to Nef at the most motif populated Nef hotspots 69–81 in yellow, and 120–130 in cyan (Figure 5). The corresponding potentially outcompeted H12 proteins were marked in pink and green, respectively. Nodes marked by multiple colors in the figure indicate those H1 proteins interacting with Nef at both hotspots in our predictions. Not all of the H1 proteins associated with outcompeted proteins are visible in this diagram due to arbitrary separation of the host protein network into distinct pathways in the KEGG database. Figure 5 indicates that our predictions point to severe remodeling of the signaling pathways by binding of HIV-1 Nef to targeted host proteins. Future work is needed to assess the predicted out competition of the proteins in this pathway.

**Table 3:** Kegg pathways statistically enriched with host proteins targeted and/or potentially outcompeted by HIV Nef.

Pathway	Count	p Value
Neurotrophin signaling pathway	47	1.33E-22
Pathways in cancer	77	3.07E-22
ErbB signaling pathway	39	8.64E-22
Natural killer cell mediated cytotoxicity	47	3.90E-21
T cell receptor signaling pathway	42	1.15E-20
Chronic myeloid leukemia	35	3.13E-20
Fc gamma R-mediated phagocytosis	38	3.51E-19
Insulin signaling pathway	43	2.00E-17
Fc epsilon RI signaling pathway	32	1.56E-16

The table lists the top 10 Kegg pathways with the lowest p value for statistical enrichment.

### Hotspots on HCV NS5A which are composed of motifs expressed by HIV Nef

**Figure 6:**
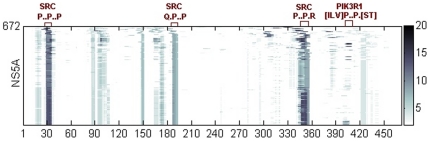
Hotspots on HCV NS5A protein composed of HIV Nef motifs. Amino acid sequence positions of motif hotspots are shown on the horizontal axis for the HCV NS5A protein. Color intensity is proportional to the number of Nef targeted proteins with enriched hotspot motifs among immediate binding partners. Motifs related to common targets of HIV Nef and HCV NS5A are shown on top in red.

High statistical enrichment of KEGG Pathway of Cancer with HIV Nef targeted and outcompeted host proteins prompted us to look at cancer association of the H1 proteins listed in Table 1. It turns out that 13 of these proteins, protein such as TP53, HCK, PIK3R1, and PSMB4, appeared in significant gene lists in various cancer types [48] and 6 (GNAO1, HLA-A, HLA-B, HLA-C, HLA-F, HLA-G) out of 13 were linked to liver cancer. Since HCV virus is commonly linked to liver cancer in United States, we focused on one HCV protein, HCV NS5A, with data suggesting that it shares host targets with HIV [16]. The study by de Chassey et al. identified presence of at least 314 protein–protein interactions between HCV and human proteins using yeast two-hybrid assay and 5 of these links included NS5A and host proteins known to bind to HIV Nef (PIK3R1, SRC). Only 21 interactions out of 314 could be validated by two different screens. None of the five host proteins cited above made the list of 21. Nevertheless, we scanned the multiple alignments of HCV NS5A with motifs computed in our study using HIV Nef as the bait and the results are shown in Figure 6. Proline rich motifs found on HIV Nef as facilitating binding to SRC is also highly spatially conserved on NS5A, with multiple sequence positions expressing the motif. The alignments also showed Nef motifs facilitating binding to host targets not known to interact with HCV NS5A; their functional roles in HCV infection have not yet been annotated. Some of the HCV NS5A sequences also express a Nef motif facilitating binding to PIK3R1 but the motif is not conserved throughout NS5A sequences, suggesting either that the motif is not essential for NS5A function or that it needs to be revised to accommodate HCV sequences [38].

## Discussion

In this study we presented a bioinformatics approach to explore the discovery of viral protein sites sticky to specific host targets using existing sequence and interactome data. Our protocol involves motif discovery on sets of sequences of proteins (viral and host) known to bind to virus targeted host proteins. We applied the methodology for the discovery of HIV Nef motifs involved in binding to thirty host targets. Our method is complementary to the method of Ofran and Rost [9] in predicting protein-protein interaction hotspots carved into sequences. Their method relies on machine learning from known interfaces between transiently interacting proteins [10]. It has high accuracy only at low coverage, and as such may be too general to apply to Nef sequence hotspots. Moreover, the method does not identify the binding partners utilizing a given hotspot on targeted protein.

The choice of Nef as the protein for illustration of our approach is appropriate due to the critical role this protein plays in HIV infectivity. Nef binding to host proteins have been experimentally studied extensively [41], [49], [50], [51], [52], [53], thus allowing us assess the accuracy of bioinformatics based predictions. Our results show viral sequence motifs shared by the host concentrate on finite number of hotspots on viral sequences and that such hotspots fall on spots with distinct 3D surface topology as well as onto relatively disordered regions of viral peptides, consistent with recent findings [3]. Structure – function relationships in HIV Nef, particularly the sequence motifs involved in post transcriptional modification, signaling and trafficking have been documented in the literature along with the identity of the interacting host protein [42], [54], [55].

Statistical comparison of our predictions of HIV Nef hotspots for binding to host proteins with corresponding experimental data yield p values smaller than 0.001 for 13 of the 30 host proteins targeted by Nef in hypergeometric test. Binding predictions for eight out of the thirteen remained significant in a more complex statistical test, the Hopkins-Skellam Index, that takes into account of the spatial distribution of predicted and experimentally annotated binding sites on HIV Nef. Statistical analysis was not possible for 6 of the 30 Nef targets due to absence of experimental data. In three other cases, binding site information contained only a few residues. In remaining cases, mismatch occurred primarily on experimentally annotated sites that fell on flexible, loop like regions of Nef. Our predictions for sites used for interacting with kinase proteins such as HCK turned out to be accurate. The kinase interaction sites we predict for HIV Nef involves motifs already linked to phosphorylation events [42]. Our predictions are on target for identifying motifs known to be interacting with protein domains SH2 and SH3 [56]. SH3 binding domain interacting compound D1 was shown to interfere with the binding of HCK to Nef but had no effect on CD4 downregulation [32], again in line with our predictions. A conformational change in 3D structure of Nef may be required for Nef interactions with the signaling proteins [57]. Taken together, the match between our predictions and experimental data provides a rationale for further exploration of viral hotspots using a variety of experimental methods and computational methods based on bioinformatics data.

Proline rich HIV Nef motifs in the core region have been previously shown to have grove like shape providing a target for drug [32]. Our results show the potential of motif discovery algorithms in identifying 3D molecular recognition sites when used on collection of sequences belonging to both host and viral proteins. A multitude of methods have been developed to assess protein surface similarities among different proteins [58]. Molecular dynamics approaches have been utilized to predict peptide-binding sites on protein surfaces [2]. Such methods can be used along with known 3D structures of viral proteins to further refine the predicted hotspots and identify whether some hotspot combinations make up topologies suitable for binding to other proteins.

Annotation of viral sequence hotspots is a first step towards prediction of bonds made and broken in cell protein networks by the invading viral proteins. Viral proteins have the potential to bridge two otherwise non-interacting proteins by binding to them using mutually exclusive sets of hot spots. Such binding events constitute making new edges in the host cell network, such as HIV Nef playing the role of linker molecule between host targeted host proteins [40], [55]. In other cases, HIV Nef uses motifs also used by host proteins to bind to SH3 domains of signaling molecules, effectively outcompeting these proteins and thereby breaking edges in the host cell network [40]. Our present approach does not uniquely identify edges broken or made but derives sets of edges statistically enriched with newly made or broken edges. KEGG cellular pathways highly enriched with host proteins identified in these sets for HIV Nef include the T cell receptor pathway, natural killer cell mediated cytotoxicity, phagocytosis, FC epsilon RI, and insulin signaling pathways. Our predictions, when combined with recent studies on topology of host networks [59] and virus host protein interactomes [24], could potentially yield useful information on the regulatory logic of host signaling pathways perturbed by viral infections.

Our study shows that the protein motifs we predict as composing HIV Nef hotspots can be utilized in the discovery of similar hotspots on the proteins of other viruses. To illustrate this potential, we scanned the multiple sequences of HCV NS5A protein for HIV Nef motifs for binding to host proteins also targeted by this HCV protein. We showed that a subset of these motifs was highly spatially conserved on the HCV protein, thus contributing to the structure-function annotation of HCV NS5A. Our approach can be used to discover and annotate protein linear motifs shared by viral and host proteins for cases with partial knowledge on virus-host protein interactomes. As such, the approach will provide a valuable tool to enrich the known collection of protein motifs with roles in protein binding events.

## Methods

### Data Acquisition

Human protein interaction data were downloaded from the Human Protein Reference Database (HPRD) [35], Release 8. The downloaded file contained binding interactions between pairs of human proteins. The HIV-1, Human Protein Interaction Database (HHPID) [17] (accessed December 2009) was used to obtain HIV Nef – host protein interactome. In addition, the research literature linked to HHPID was screened to prepare summaries of experimental data concerning motifs and sequence segments involved in host - Nef protein interaction events. The HIV-1 Sequence Database [60] for subtypes A, B, C, and D (2008 version) was used to download multiple protein alignments of the HIV protein Nef as the reference dataset of Nef sequences in this study. The Eukaryotic Linear Motif (ELM) resource was used to annotate HIV Nef protein sequences with ELMs. The same set of sequences was also screened for the motifs discovered in the present study.

### Dataset preparation

The HHPID database partitions annotated interactions between HIV and host proteins into different categories. In this study, 42 human proteins in HHPID were identified as participating in binding, phosphorylation, and cleavage interactions with HIV Nef (H1 proteins). All three of the chosen interaction types involve direct binding of Nef to human proteins while other interaction categories listed in HHPID do not imply direct physical interaction between the two protein (e.g. *downregulates*, *upregulates*, *activates*, etc.). Thirty out of the forty-two H1 host proteins had at least ten direct binding partners (H2s) (Table 1). We limited our analysis to H1 proteins with at least ten H2s because the motif discovery tool SLiMFinder [28] used in this study works best for sets of protein sequences numbered from tens to hundreds.

In motif discovery, we took the amino acid sequences of H2 proteins for a given H1 and added to this set randomly chosen HIV Nef sequences equal in number to the larger integer closest to 10 percent of the number of H2s. For H1s with ten to twenty binding partners, this amounted to two HIV Nef sequences. Addition of Nef sequences into sequences used for motif discovery eliminated a large number of the motifs discovered on H2 proteins but not present on Nef sequences. In total, 30 datasets of protein sequences were created, one for each H1 with at least ten partners for the discovery of motifs potentially involved in the binding interactions of Nef and its host protein partners.

Protein sequence datasets thus prepared were fed into SLiMFinder [28] for the discovery of motifs ranging from 3 to 10 amino acids in length. To reduce the rate of false positive discovery, SLiMFinder first detects homologous sequences within the input dataset using BLAST [61] to determine their evolutionary relationship and form unrelated protein clusters (UPC), which are defined such that no protein in a UPC has a relationship with any protein in another UPC [28]. The Blast e-value used in this tool was set to 1E-28. In the next step, SLimFinder searches for motifs in all proteins and then weights results according to the evolutionary relationship for the proteins containing the motif. It also discovers patterns (motifs) with semi-conserved (wildcard) positions and results are presented using regular expressions. Other parameters for motif discovery in SLiMFinder were set to the default values in the tool manual.

Motifs identified by SLiMFinder for a given H1 were then matched to human protein sequences to eliminate abundant motifs. Motifs present in more than 30 percent of HPRD proteins were filtered out, as ubiquitous motifs have been shown to be poor predictors of HIV- host interactions [23]. The p value for statistical enrichment of a motif in H2 proteins for a given H1 compared against their background expression in HPRD in hypergeometric test was set equal to 0.005. Motifs that satisfied these criteria were then checked to assure that they were expressed at least by 20 percent of H2 proteins associated with the given H1 protein. Another requirement for further annotation of the discovered motifs is their conserved presence on the HIV Nef sequences. Motifs that were not present on at least 70% of all subtypes of the Nef protein sequences were removed. Therefore, the final list of motifs contained those motifs overrepresented on H2 proteins, not abundant on HPRD and largely conserved on existing HIV Nef sequences. Motifs in the final list were compared to the latest list of ELMs using CompariMotif [27]. Similarity score of 2.7 was used as the cut-off to detect similar patterns of motifs.

### HIV Nef sequence hotspots for crosstalk with host proteins

The H2 motifs that passed the processing described above were projected onto HIV Nef protein sequences. Many of these motifs partially overlapped on the sequence. These motifs were clustered automatically based on their location on the Nef sequence using an iterative merging algorithm. At the beginning, each motif is considered as a cluster. Two clusters are merged if the sum of the mismatch between their beginning and ending positions (on Nef) is less than six amino acids. Merging was accomplished iteratively through the motif list until no cluster changed. Each such cluster was deemed a hotspot and was labeled by the start and the end sequence locations on Nef. These hotspots contained motifs potentially facilitating binding events with multiple H1 proteins. Such H1 proteins were identified by statistical enrichment of a motif in the hotspot among binding partners of H1. H12 proteins were identified by requiring members (subset of H2) to express at least one motif clustered on a Nef hotspot. The H12 proteins constituted host proteins potentially outcompeted by HIV Nef.

### Statistics for comparison of predicted binding sites with experimental data

The gene symbol for each of the thirty H1 proteins was fed into the NCBI Gene Source in order to identify experimentally determined binding sites on HIV Nef. The *HIV-1 protein interaction* link in the source presented brief summaries of articles describing an interaction of Nef with the host protein coded by the gene. We used these summaries to identify the Nef sequence segments known to bind to H1 proteins. In cases where the summaries did not present a binding site but identified a binding motif, we identified the binding site by reading the research article. We did not use the directed mutagenesis data showing a single phenotype altering mutation on Nef as the link of such data to prediction of binding sites is not yet clear. Overall, experimental data on Nef binding sites to H1 proteins is far from complete and therefore, we refrained from a more detailed curation than conducted as described.

In order to assess statistical significance of the match between prediction and experiment, we projected experimentally verified binding sites and predicted motifs for each H1 onto the HIV Nef sequences. These sites and motifs were spatially conserved on approximately 50 percent of the sequences. We used two different statistical test to assess significance. First, we used the lengths of Nef, predicted binding sites, experimental binding sites and their intersection in hypergeometric test. Secondly, we utilized the spatial information presented in Figure 2 in Hopkins-Skellam Index computations [62] to estimate the significance of the intersection between predicted and experimental Nef binding sites for each of the thirty H1 proteins. This method tests whether the predicted sites are closer to the known experimental binding sites then randomly generated sites with same characteristics as the predicted sites. In this approach, we first calculated distances from the centers of experimental binding sites to nearest predicted sites (Dp) for each of the H1 proteins under consideration. Then we generated 10,000 random sites with the same characteristics (number and length of sites) as predicted sites and for each such randomly generated binding site cluster computed the distance (Drt) from the experimental binding site (target site). Similarly, we have generated 10,000 random sites with characteristics of the target site and computed the distances from predicted to random target (Dpr) and from random predicted to random target (Drr). We called the ratio (Drt/Dpt) as HSI and the ratio (Drr/Dpr) as rHSI. The fraction of the permutation events where rHSI<HSI was set equal to the p value. We assumed significance for p<0.05. The method previously described here has recently been used to assess the randomness of the distribution of epitopes for cytotoxic T lymphocytes on HIV proteins [37].

## Supporting Information

Table S1 (DOCX)
