# Supplementary material for: Sequence- and Interactome-Based Prediction of Viral Protein Hotspots Targeting Host Proteins: A Case Study for HIV Nef
Source: PLoS One. 2011 Jun 28;6(6):e20735. doi: 10.1371/journal.pone.0020735 (PMC3125164; doi:10.1371/journal.pone.0020735)
Supplement: Table S1 — (DOCX) [file pone.0020735.s001.docx]

**Table S1.** Motif clusters along the sequence of HIV Nef and their correspondence to eukaryotic linear motifs (ELMs). The table presents the list of HIV Nef motifs enriched among the immediate neighbors of human proteins targeted by Nef. The columns identify the Nef sequence segment occupied by the cluster, regular expression, and start end positions of each motif within a hotspot, the host protein predicted to be interacting with the motif, and the most similar ELM to the motif pattern.

| **Cluster** | **Motif Pattern** | **Start** | **End** | **Associated h1s** | **ELM** |
| --- | --- | --- | --- | --- | --- |
| 1_8 | [IMV]..K.[GS][HK] | 1 | 8 | AP1M1 | - |
| 13_19 | W.{1,2}A.{0,2}E | 13 | 19 | VAV1 | - |
| 26_35 | [AS]..[GS]..[AGS].S | 26 | 35 | AP1M1 | LIG_WH1 |
| 31_37 | [GS][AS].[ST].[DE] | 31 | 37 | CD4 | - |
| 37_42 | L.K.G | 37 | 42 | TP53 | - |
| 44_48 | [ST][ST].N | 44 | 48 | FYN | - |
| 66_72 | [DE][LV][GS]F | 66 | 70 | PAK2 | - |
|  | [DE].[GS][FH].[LV] | 66 | 72 | AP1M1 | - |
| 69_85 | F.[FILV]..Q | 69 | 75 | PAK2 | - |
|  | [FV]..[KR]P.[IV] | 69 | 76 | AP1M1 | - |
|  | P..P..P[FILV] | 70 | 78 | HCK | LIG_SH3_1 |
|  | P.{0,2}P.{0,2}P.{0,2}P | 70 | 80 | MAPK1, FYN, HCK, LCK, SRC, PIK3R1 | LIG_SH3_1 |
|  | V.P..P | 71 | 77 | ARF1 | LIG_SH3_1 |
|  | [HKR]P..P | 72 | 77 | FYN, VAV1, SRC | LIG_SH3_1 |
|  | P.VP | 73 | 77 | FYN, HCK, VAV1 | LIG_SH3_1 |
|  | P..P.[HKR] | 73 | 79 | FYN, VAV1 | LIG_SH3_2 |
|  | P..P.R | 73 | 79 | MAPK1, FYN, HCK, VAV1, SRC, PIK3R1 | LIG_SH3_2 |
|  | Q.P.[HKR] | 74 | 79 | FYN | - |
|  | Q.P..P | 74 | 80 | PAK2, FYN, HCK, LCK, SRC | LIG_GYF |
|  | PL.P | 75 | 79 | ARF1 | LIG_GYF |
|  | [FLV]P..P.T | 75 | 82 | HLA-A | LIG_SH3_1 |
|  | P.[KR]P | 76 | 80 | VAV1 | LIG_SH3_1 |
|  | P.[HKR]P | 76 | 80 | VAV1 | LIG_SH3_2 |
|  | L.P.T | 76 | 81 | GNB2L1 | - |
|  | P[LMV].P.[ST] | 76 | 82 | HCK | LIG_SH3_1 |
|  | P..P.T | 76 | 82 | PAK2, FYN, HCK, HLA-A | LIG_SH3_1 |
|  | P..P.[ST]..[AG] | 76 | 85 | HLA-A | LIG_SH3_1 |
|  | P..P.[ST]..[AGS] | 76 | 85 | MAPK1, CALM1 | LIG_SH3_1 |
| 80_92 | [LM][ST][FY]..[AG]..[FIL] | 80 | 89 | PAK2 | - |
|  | [HK]..[FLV].L..[FY] | 83 | 92 | ARF1 | - |
| 85_96 | [AS]..[FL]..[FLM]..E | 85 | 95 | MAP3K5 | LIG_BRCT_BRCA1_1 |
|  | [DE]L[GS][FH] | 87 | 91 | PAK2 | - |
|  | [DE]..[FHY][FY] | 87 | 92 | FYN | LIG_WH1 |
|  | [DE]L..[FL]L | 87 | 93 | LCK | TRG_LysEnd_APsAcLL_1 |
|  | D..[FH][FL][LV] | 87 | 93 | HCK | TRG_LysEnd_APsAcLL_1 |
|  | [DE]L..[FIL][IL] | 87 | 93 | MAPK1 | TRG_LysEnd_APsAcLL_1 |
|  | [DE][ILM]..[FIL]..E | 87 | 95 | RAF1 | - |
|  | L..FL | 88 | 93 | HLA-A | - |
|  | L..[FIL]L[KR] | 88 | 94 | VAV1 | LIG_NRBOX |
|  | [FIL]..[FI][IL]..[KR] | 88 | 96 | HLA-A | - |
|  | [FIL]..F[IL]..[KR] | 88 | 96 | HLA-A | - |
|  | [IL]..[FY]L..[HKR] | 88 | 96 | ARF1 | MOD_TYR_DYR |
|  | [GS].[FIL][LV]K | 89 | 94 | ARF1 | - |
|  | [FH]FL..[KR] | 90 | 96 | HLA-A | - |
| 90_102 | [FHY]..K.[KR]..[FLV] | 90 | 99 | MAP3K5 | LIG_MAPK_1 |
|  | F.{0,2}L.{0,2}K | 91 | 94 | FYN | - |
|  | F.KE | 91 | 95 | ARF1 | - |
|  | FL..K | 91 | 96 | HLA-A, VAV1 | - |
|  | [FWY]..E..G..G | 91 | 101 | PAK2 | - |
|  | L..K.G | 92 | 98 | AP2B1 | - |
|  | [GS].L[DE].[FL] | 96 | 102 | AP1M1 | - |
| 108_113 | Q.{1,2}I.{0,2}D | 108 | 113 | ARF1 | - |
|  | [DE][ILMV][LV][DE] | 109 | 113 | AP2B1 | - |
|  | I.{0,2}L.{0,2}D | 110 | 113 | VAV1 | - |
| 111_117 | [LV].[IL][WY].Y | 111 | 117 | PAK2 | - |
|  | [DE].[FWY].[FY] | 112 | 117 | RAF1 | - |
| 118_126 | Q.{0,2}F.{0,1}D | 118 | 124 | AP2B1 | - |
|  | QG.[FILV]P | 119 | 124 | PAK2 | - |
|  | Q..[FY].D | 119 | 125 | AP1M1 | - |
|  | Q..[FWY]..[FWY] | 119 | 126 | CALM1 | - |
|  | Q..[FHY]..[FWY] | 119 | 126 | CALM1 | - |
|  | [GS][FY][FI]P | 120 | 124 | AP1M1 | - |
|  | G[FWY][FIV]P | 120 | 124 | PAK2 | - |
|  | G[FY][FI]P | 120 | 124 | PAK2 | - |
| 121_132 | [FY]..[DE][FWY] | 121 | 126 | MAP3K5 | - |
|  | [FHY]..[DE]..N | 121 | 128 | AP2B1 | - |
|  | P..Q.[FY] | 123 | 129 | GNB2L1 | - |
|  | [DE].Q.[FWY] | 124 | 129 | FYN | LIG_CAP-Gly_1 |
|  | [DE]..N.[ST] | 124 | 130 | FYN, LCK | - |
|  | [FWY].N.[ST] | 125 | 130 | CD4 | LIG_SH2_GRB2 |
|  | W.{0,2}Y.{1,2}G | 125 | 132 | CD4 | - |
|  | N[FHY][ST] | 127 | 130 | GNB2L1 | - |
| 130_138 | P.P..[HKR] | 130 | 136 | FYN, HCK | LIG_GYF |
|  | P.P..[HKR].P | 130 | 138 | HCK | - |
|  | P..[HKR].P | 132 | 138 | MAPK1, FYN | - |
| 136_141 | [FY]P..[FHY] | 136 | 141 | AP1M1, PAK2 | - |
|  | [FY]P..[FY] | 136 | 141 | AP1M1 | - |
|  | [FWY]P..[FY] | 136 | 141 | LCK | - |
| 139_145 | T.{0,2}F.{0,1}W | 139 | 143 | GNAO1 | - |
|  | [FW][GS]..[FWY] | 140 | 145 | MAP3K5 | - |
| 160_169 | N..[IL].P | 163 | 169 | HLA-A | - |
| 200_206 | [FHY]P..[FY] | 200 | 205 | PAK2 | - |
|  | PE.{0,1}Y | 201 | 204 | HLA-A | - |
|  | [DE][FY][FWY] | 202 | 205 | AP2B1, RAF1 | - |
|  | [DE][FWY][FY] | 202 | 205 | AP1M1 | - |
|  | [DE][FY][FY] | 202 | 205 | AP2B1, RAF1 | - |
|  | [FWY][FY][HK] | 203 | 206 | VAV1 | LIG_WH1 |
